# Thematic Research network for emergency and UnScheduled Treatment (TRUST): scoping the potential

**DOI:** 10.1186/1471-227X-8-2

**Published:** 2008-01-30

**Authors:** Julie Peconi, Helen Snooks, Adrian Edwards

**Affiliations:** 1 Centre for Health Information, Research and Evaluation (CHIRAL) School of Medicine, Swansea University, Singleton Park, Swansea, Wales, SA2 8PP, UK; 2 Professor Adrian Edwards, Department of General Practice, Centre for Health Science Research, University Hospital of Wales, Wales, CF14 4YS, UK

## Abstract

**Background:**

To identify the benefits of a network in emergency and unscheduled care research, a six week scoping study was undertaken. Objectives were to: draw together stakeholders; identify and prioritise research topics; identify sites for recruitment to studies; and agree a research strategy for a network.

**Methods:**

A workshop was held to discuss and agree a research strategy based on results from four activities: visits to established research centres in emergency and unscheduled care; a literature overview; interviews with stakeholders in a GP out-of-hours service; and an exploration of the potential for routine data to support research in emergency care.

**Results:**

Participants attended the workshop from user groups, primary care, the ambulance service, social care, the national telephone based health helpline, the Welsh Assembly Government and the academic sector. Site visits identified opportunities for collaboration. Gaps in knowledge were identified concerning the effectiveness of alternative models of emergency care delivery. Interview data highlighted a lack of evidence related to the quality of out-of-hours provision of primary care. The All Wales Injury Surveillance System (AWISS) was found to offer the potential to use routine data to support quantitative studies in emergency care. Three key issues emerged across all activities: working across boundaries; patient involvement; and triage.

**Conclusion:**

The study included views from patient, provider, policy and academic perspectives and built the case for a research network in emergency care. Now funded, TRUST (Thematic Research network for emergency and UnScheduled Treatment) will allow the development of research proposals, building of research teams and recruitment of sites and patients both in Wales and across the UK. It aims to address the imbalance between investment and research in this area and help support provision of 'the right care to the right people at the right time'.

## Background

Thematic research networks are perceived to be one way of enhancing capacity for research and development to provide an evidence base for healthcare. Research networks have been established in England through the UK Clinical Research Collaboration (UKCRC), a partnership of the major stakeholders influencing clinical research in the UK. The UKCRC aims "to establish the UK as a world leader in clinical research, by harnessing the power of the NHS" [1]. It has developed the UK Clinical Research Network (UKCRN) to support clinical research and to facilitate the conduct of randomised prospective trials of interventions and other well designed studies [2]. The UKCRN supports six topic-specific clinical research networks in England: in cancer, dementias and neurodegenerative diseases, diabetes, medicines for children, mental health and stroke.

Against this backdrop, in February 2005, the Wales Office of Research and Development for health and social care (WORD) issued a call for proposals for 'scoping' studies, to identify the benefits of establishing thematic research networks in Wales. Scoping study funding was intended to enable 'preparatory activities to help individuals and groups form new networks, or develop existing networks and consolidate their development planning'. The Emergency and Unscheduled Care Thematic Research Network (TRN) scoping study was one of twenty-six, commissioned to be undertaken within a short period of six weeks. Following submission of the scoping studies, a further call was issued, for full thematic research networks.

In December 2005, based on results of the scoping study activities, TRUST: Thematic Research network for emergency and UnScheduled Treatment was one of nine networks funded by WORD for an initial period of three years. Although local emergency care research networks have been established) [3] and the importance of a whole systems approach in emergency care recognised [4], this is the first time in the UK that a national network in emergency care research has been funded and the opportunities it presents are significant.

With increasing demand on all emergency services in the UK and other developed countries [5,6], and evidence that a substantial proportion of emergency ambulance callers and Emergency Departments (ED) attenders do not clinically need the service offered [7-9], it is vital to develop and evaluate the system that delivers care to those who ask for an immediate response. In this paper we use the term unscheduled care to describe care that is provided (or demanded) without warning or planning. The term covers a range of clinical and social conditions and needs, from life-threatening emergencies to requests for information or assistance for problems that may not be clinically urgent.

In the UK there are many points of access to unscheduled care; the emergency ambulance (999) route, national telephone health helpline (NHS Direct), general practitioner (GP) surgery or out-of-hours service, ED, Minor Injuries Units and community pharmacists. Patients call or attend providers with a wide range of healthcare needs for treatment, transportation, advice and information. Patients need to be triaged so that those with urgent or life-threatening clinical problems can be identified for an immediate response, and those with less urgent conditions can be offered care that is appropriate to their needs without wasting National Health Service (NHS) resources. 'Our health, our care, our say', the policy document recently published by the UK Department of Health, highlights the need to improve patient experience and significantly reduce unnecessary hospital admissions through a new strategy for urgent care [9]. Implementation of effective prevention strategies and increasing the ability of patients to manage their perceived 'emergencies' at home or with the help of community based facilities such as the pharmacist, fit with the current emphasis on shifting resources and care from the secondary to the primary sector [10].

The emergency and unscheduled care sector is undergoing rapid change. New models of care are being implemented through the ambulance service (e.g. paramedic practitioners with extended skills training), NHS Direct, a 24 hour nurse-led telephone based healthcare advice and information line (e.g. handling non-serious 999 calls), Minor Injuries Units and Walk-in Centres. New primary care contracts in general practice and community pharmacy also provide the opportunity to change the pattern of care delivered. A new contract in 2003–4 allowed general practitioners to 'opt out' of their traditional 24 hour availability. Almost all took up this option enabling a completely new provision for out-of-hours primary care commissioned by Primary Care Organisations (PCOs). Currently a patchwork of deputising-derived services, GP co-operatives, NHS Direct-linked services, and Trust-based contracts are provided. These new models of care have yet to be fully evaluated to understand the impact on patient care and the NHS through, for example, changes in demand for other services.

Still further changes are anticipated in the next few years. Among these, review and revision of skill mix and professional provision are likely. Greater integration with NHS Direct and availability of advice and information, often online or via digital television, is also probable. Yet many of these changes do not build on an evidence base of safety and effectiveness and formal evaluations are rare, with the opportunity to learn from new initiatives missed.

The aim of this scoping study was to explore the potential benefits of the development of a research network for emergency and unscheduled care both in Wales and beyond. Its objectives were to draw together key stakeholders in emergency and unscheduled care, identify partner sites for recruitment to trials and other studies, identify research topics of priority and agree an overall research strategy for a network. This paper describes the development and rationale of the network through the scoping study and highlights the role the network can now play in enhancing research and development to meet the rapidly changing context of the emergency and unscheduled care field.

## Methods

The scoping study was led by HS from the School of Medicine, Swansea University. The study involved one dedicated Research Officer (JP) and included participants from across Wales and specialist collaborators in England. The research team included co-applicants from all sectors that contribute to the provision of emergency/unplanned care: ED, GP out-of-hours, the Welsh Ambulance Service Trust, NHS Direct Wales, and social care. Within each sector representation was gained from service providers, policy makers, academics and service users.

During the short study period the team employed four methods of data collection: site visits to key centres of emergency and unscheduled care research in the UK, a literature overview, interviews with stakeholders and an exploration of the use of routine data to support research studies in emergency care. Results from these four strands were then fed into a one-day workshop which focused on identifying the benefits of establishing a research network in emergency and unscheduled care in Wales, and research strategies and priorities for such a network.

### Site visits

Site visits were made to key centres of emergency and unscheduled care research in the UK: the Medical Care Research Unit at Sheffield University and the Centre for Primary Health Care Studies at the University of Warwick. These sites were selected due to their track records of undertaking and publishing research in this field and their strong links to policy makers in the UK through contractual arrangements with the department of health. Discussions were based on a semi-structured interview guide, and centred on research programme plans, overlapping research interests and opportunities for collaboration.

### Literature overview

A Medline search on alternatives to hospital/emergency care from 1984 onwards was undertaken. Search terms included '(alternative) and (hospital)' and '(alternative) and (emergency) and (care)'. Due to time constraints, results were then limited to systematic reviews, meta-analyses and randomised controlled trials.

### Interviews with stakeholders

A series of semi-structured interviews were held with stakeholders as key informants in one GP out-of-hours service in Wales. During interviews, respondents were asked about their views concerning current service provision, current and anticipated research needs, service development and potential benefits of a network for research in emergency and unscheduled care. Interviews were conducted by AE and field notes were taken. Results were analysed and synthesised according to existing or potential priority areas for research and capacity building needs in the sector.

### Exploration of routine data

Discussions took place with the lead for the All Wales Injury Surveillance System (AWISS) to explore the potential for routine data to support research in emergency and unscheduled care. AWISS is a computerised system designed to collect and collate information on injuries treated in ED in Wales. During the study, data were sought from EDs across Wales in order to run analyses to determine factors contributing to increased demand for ED care.

### The workshop

An invitation to the workshop was extended to all those involved in the scoping study and other commissioned scoping studies with overlapping interests and concerns. Other key stakeholders were invited, including representatives of patient groups and additional researchers in emergency/unscheduled care. All invitees were encouraged to bring colleagues who they felt could also contribute to the formation of the network. Presentations were given on the preliminary findings from the four strands above. Delegates were then split into discussion groups to identify research priorities and network strategies on the key themes that had emerged from the presentations. Delegates chose which discussion to attend based on their area of expertise and personal interest. Each group was asked to consider their respective theme in terms of a research strategy, key research questions and priorities to be addressed through the network. Key points from each discussion were then fed back to the full group in a plenary session and other delegates were invited to comment on and/or add to the results.

## Results

### Site visits to centres of research excellence in emergency and unscheduled care

The Scoping Study lead (HS) and Research Officer (JP) visited the Medical Care Research Unit in Sheffield and the Centre for Primary Health Care Studies at the University of Warwick. The visits enabled the identification of opportunities for collaboration. The programmes in Sheffield (in part core-funded by the Department of Health) and Warwick focus on research concerning the system of emergency care provision, rather than on individual treatments or elements of that system. Partners at the collaborating sites were in agreement that the funding of a network in emergency care in the UK could be valuable in allowing the development of programmes of research in this area. They agreed that the proposed network could help to develop capacity and collaboration in emergency and unscheduled care research, allowing research funding to be drawn into the area from generic sources aimed at improving quality of care and cost-effectiveness of service delivery. In addition, a useful function of the network was felt to be the enhanced opportunity for recruitment of sites and patients to randomised controlled trials.

### Literature overview

Current research uncovered through the literature overview included work on novel forms of home care, particularly involving nurses or therapists in forms of care traditionally undertaken by doctors; on the development of definitions and standardisation of methods in an emergency care setting and on specific problems such as triage and protocols for training. One recent systematic review focussing on reducing attendances and waits in EDs was reviewed although much of the literature identified was concerned with opinions and causes, rather than evaluations of innovations to reduce these waits and attendances.

However, overall, the overview revealed that there is a lack of high quality published research – particularly randomised controlled trials – on the topic of the provision of alternative models of current emergency and unscheduled care. Gaps in current knowledge concerning effectiveness of alternative models of emergency care delivery were identified as well as the need for the development of definitions and protocols.

### Interviews

Eighteen semi-structured face to face interviews were carried out with stakeholders (including GPs, policymakers and directors) in one GP out-of-hours cooperative in Wales, and the service and policy leads in this area, to identify key research issues in out-of-hours provision. Specific aspects highlighted as requiring review or evaluation included: the effectiveness of call handling, the efficacy of nurse and doctor triage in preventing home visits and consultations and evidence to support the newly introduced national four hour waiting time targets in ED. Difficulties in engaging consumers in setting priorities, integrating patients' experiences of the services into subsequent development of the services, managing demand and communication with other sectors (e.g. palliative care) were also identified as topics requiring further research.

### Routine data exploration

Although initial analysis of data from one Trust in South Wales highlighted possibilities for understanding patterns of changing demand, retrieval of data from hospitals did not prove possible to achieve within the scoping period. The study concluded that, if full participation could be achieved, routine data gathered through the All Wales Injury Surveillance System (AWISS) could offer the potential to support studies with a wide range of designs, including: randomised controlled trials, cohort studies, ecological and multi-level studies, modelling, time series analyses, cross-sectional and database analyses as well as multi-agency studies.

### The workshop

The network workshop was held on 19^th ^April 2005 in Swansea, with twenty-five participants from user groups, primary care, the ambulance service, social care, NHS Direct Wales, the Welsh Assembly Government and the academic sector. Although representatives from ED expressed interest, due to work constraints, no one from this key sector attended.

There were approximately equal numbers in each discussion group with representatives from across disciplines in each group. Discussions focused on three key issues that had emerged from the presentations:

1) working across service boundaries to provide appropriate unplanned care

2) involving patients in the planning and delivery of emergency and unscheduled care

3) triage: how to 'sort' patients so that the right care is offered to the right patient at the right time

Delegates identified clear gaps in evidence that need addressing to allow planning and implementation of new services, particularly concerning the effectiveness of complex interventions and their wider or halo effects; triage; issues of access; patients' views and priorities; and drivers for demand. It was also noted that, whilst research with practical and relevant outcomes are a priority for the network, the development of methods for evaluation and indicators of quality should not be neglected.

There was consensus that a thematic research network in emergency and unscheduled care could draw together stakeholders with a range of skills and perspectives to develop and enhance research in this field, in order to address a current mismatch between investment in service development and reconfiguration and the evidence base for new service delivery arrangements.

## Discussion

This scoping study confirmed the dearth of high quality research in emergency and unscheduled care and highlighted the potential for a national thematic research network to help address the lack of robust evidence in this area. Given the short time frame involved, the exercise set out to identify key problems facing emergency care research today, in order to inform the development of a network, its activities and focus. The study was necessarily limited in its scope by the short term nature of funding. Within six weeks activities were undertaken that were felt to be most useful in assessing the need and potential benefits of a research network in emergency and unscheduled care. We did not quantify these benefits or assess costs.

Opportunities for collaboration with some English centres of excellence were identified through similar programmes of research although further engagement of existing emergency care groups across the UK and internationally is necessary. The network would also be able to assist in recruitment of sites and patients for studies and offer a means of effective consultation with a range of key stakeholders. This initial series of interviews with stakeholders in GP out-of-hours service providers, the analysis of routine data and the workshop revealed further questions and research priorities. Scoping study activities brought together key players and stakeholders in emergency and unscheduled care, strengthening commitment to the proposed network.

Thematic networks can work, but need to focus their activities and build capacity to achieve critical mass [11]. Drawing together interested parties for discussions only at a 'talking shop', or providing basic research skills training is not enough to justify investment in the infrastructure. The network will need to develop capacity and systems for the maintenance and expansion of research activities over time. It will then need to add value through bringing in research money; providing focused training and mentorship in research skills and through helping to publish research findings that will contribute to building the evidence base priority areas.

Researchers, providers of care across the sectors of ED, the ambulance service, primary care, NHS Direct Wales, social care and policy makers from across Wales were brought together through this scoping study. Medical, nursing, paramedical, academic and other professional and patient groups were represented. It is the first time in Wales that these groups have been brought together across the emergency care system, to focus on research issues in this way. The opportunity for development of a programme of research to meet the perspectives and needs of the participating sectors was recognised as unique and was highly valued.

The size of Wales (manageable distances and structures for collaboration), interest levels across sectors, existing research strength in the area and priority for policy makers support the development of the network and highlight the potential benefits it could bring to this field. The current research infrastructure in Wales, with the establishment of the Clinical Research Collaboration Cymru, of the UK Clinical Research Collaboration, and the forthcoming Research Professional Network, a network of dedicated professionals to support research [12], will be fundamental to the success of TRUST. Investment in the network will now allow initial links to be strengthened and built upon through application for research funding for projects, and eventually a programme of research that would include infrastructure funding to support the network both in Wales and further a field.

## Conclusion

A research network focussing on emergency/unplanned care, now funded, is ideally placed to address the imbalance between investment in innovations and underpinning research evidence to the benefit of patients and the wider NHS.

TRUST: Thematic Research network for emergency and UnScheduled Treatment aims to build on the existing area of research strength in this field and of co-applicants, specifically where there is a lack of evidence to underpin current developments. It aims to attract new research funding into the field of emergency and unscheduled care and will contribute to the development of research proposals, allow efficient building of teams and recruitment of research sites and patients. If successful, TRUST will build research capacity in emergency and unscheduled care in Wales and beyond, and will contribute to the required evidence base to support the provision of the *'right care to the right people at the right time'*.

## Competing interests

The author(s) declare that they have no competing interests.

## Authors' contributions

HS designed the study, brought together participants, contributed to data collection and led the writing of the scoping study report on which this article is based. JP contributed to data collection, wrote sections of the original report and drafted this article. AE led the interviews with stakeholders in the GP Out-of-hours service and wrote this section of the final report. All authors contributed to refining the paper through revising and commenting on earlier drafts.

## Pre-publication history

The pre-publication history for this paper can be accessed here:


